# Postoperative brain abscesses and infections after neurosurgery: Clinical characteristics, risk profiles, microbiological findings, and longitudinal DWI and ADC MRI analysis

**DOI:** 10.1016/j.bas.2026.106093

**Published:** 2026-05-15

**Authors:** Biyan Nathanael Harapan, Antonia Clarissa Wehn, Luis Kuschel, Janine Herrmann, Béatrice Grabein, Robert Forbrig, Florian Ringel, Michael Schmutzer-Sondergeld

**Affiliations:** a Department of Neurosurgery, LMU University Hospital, LMU Munich, Marchioninistraße 15, Munich, 81377, Germany; b Department of Clinical Microbiology and Hospital Hygiene, LMU University Hospital, Campus Großhadern, Marchioninistraße 15, Munich, 81377, Germany; c Institute of Neuroradiology, LMU University Hospital, LMU Munich, Marchioninistraße 15, Munich, 81377, Germany

**Keywords:** Brain abscess, Empyema, Postoperative infection, Infection, Epidural infection, Subdural infection

## Abstract

**Introduction:**

Postoperative surgical site infections (brain abscesses and empyemas) are serious complications after neurosurgical procedures and differ from primary abscesses regarding risk factors, pathogen spectrum, and clinical course.

**Research question:**

What are the clinical characteristics, laboratory and microbiological profiles, and longitudinal DWI/ADC imaging changes of postoperative surgical site infections, and which factors are associated with patient outcome?

**Material and methods:**

This retrospective single-center study included patients treated for postoperative surgical site infections between 2014 and 2024. Demographics, surgical methods, comorbidities, neurological scales (GCS, KPS, GOS, mRS), laboratory markers (CRP, leukocytes, IL-6), antibiotic therapy, and microbiology were analyzed. Serial MRI with DWI and ADC quantification was evaluated preoperatively and at three postoperative time points (within 7 days, 4–12 weeks, and last follow-up).

**Results:**

113 patients (55 males, 58 females) had a mean age of 56.8 ± 15.1 years and underwent 361 surgeries (3.2/patient); bone flap removal was necessary in 73.5%. The presence of a diffusion restriction decreased from 60.4% preoperatively to 16.1% at final follow-up, accompanied by a significant postoperative ADC increase. Infections most frequently followed surgery for glioma (37.2%) and meningioma (22.1%). Staphylococci and Gram-negative bacteria predominated; polymicrobial infections were frequent. Mean total antibiotic duration was 48.0 ± 25.9 days (epidural infections) and 44.1 ± 19.3 days (subdural infections) (p=0.4).

**Discussion and conclusion:**

Postoperative surgical site infections exhibit distinct microbiology and risk patterns. Combined surgical revision and targeted prolonged antibiotics are essential. Longitudinal DWI and ADC quantification serve as objective imaging biomarkers for monitoring abscess activity and treatment response.

## Highlights

• Postoperative infections show distinct microbiological patterns and risk profiles.
• Serial MRI demonstrates progressive decline of diffusion restriction after treatment.
• ADC values show a consistent longitudinal increase across postoperative follow-up.
• Subdural infections associate with elevated CRP levels and polymicrobial detection.
• Recurrence dynamics differ significantly between epidural and subdural infections.

## 1 Introduction

Postoperative intracerebral abscesses and epidural/subdural empyemas represent severe and challenging complications following neurosurgical procedures (Lange et al., 2018). Although advances in sterile technique, perioperative antibiotic prophylaxis, and microsurgical methods have markedly reduced their incidence, postoperative infections of the central nervous system remain associated with substantial morbidity and mortality (Zhu et al., 2024). These infections can arise days to months after the index operation and may involve the surgical cavity, epidural or subdural spaces, or deeper brain parenchyma (Dashti et al., 2008).

Unlike primary brain abscesses, which typically result from hematogenous spread or contiguous infection from otogenic or sinus sources, postoperative surgical site infections usually occur secondary to direct inoculation during surgery or as a consequence of wound dehiscence, cerebrospinal fluid leakage, or the presence of foreign material such as bone flaps, implants, or dural substitutes (Carone et al., 2025). The underlying pathophysiology is multifactorial, influenced by both host and procedural factors, including the type and duration of the initial operation, immunosuppressive therapy, adjuvant radio- and/or chemotherapy, and systemic comorbidities such as diabetes or obesity (Guo et al., 2021).

The microbiological spectrum of postoperative surgical site infections also differs from that of primary brain abscesses, with a predominance of *Staphylococcus aureus*, *Staphylococcus epidermidis*, and Gram-negative pathogens, and frequent polymicrobial findings (Dashti et al., 2008). Accurate identification of causative organisms through surgical sampling is essential for guiding targeted antimicrobial therapy.

Management requires close interdisciplinary collaboration among neurosurgeons, infectious disease specialists, and neuroradiologists. Timely surgical debridement or abscess evacuation combined with prolonged, pathogen-directed antibiotic therapy remains the cornerstone of treatment (Dhar and Pal, 2021). However, despite these principles, evidence on clinical outcomes, recurrence rates, and prognostic factors in postoperative brain abscesses and empyemas remains limited.

Magnetic resonance imaging (MRI) plays a central role in the diagnosis and follow-up of postoperative infections. While diffusion-weighted imaging (DWI) and apparent diffusion coefficient (ADC) mapping are established for differentiating abscesses from other postoperative changes (Reddy et al., 2006; Chang et al., 2002; Berndt et al., 2018; Schwartz et al., 2019), most available studies are limited to a small number of follow-up examinations and short-term imaging courses rather than systematic longitudinal assessment (Cartes-Zumelzu et al., 2004; Moradi et al., 2025; Cui et al., 2024). Systematic longitudinal MRI analyses – particularly those incorporating quantitative ADC measurements, diffusion dynamics, and temporal signal evolution across different sequences – remain scarce. Likewise, volumetric or sequence-specific biomarkers that may reflect treatment response, resolution, or risk of recurrence have not been sufficiently investigated in postoperative abscess populations.

Against this background, the present retrospective study investigates patients treated for postoperative brain abscesses or surgical site infections at the LMU University Hospital between 2014 and 2024. By integrating longitudinal MRI follow-up – including detailed analysis of DWI and ADC dynamics – with clinical, microbiological, and laboratory data, this study aims to improve the understanding of postoperative infection evolution, identify imaging correlates of treatment response, and contribute to more refined risk stratification and outcome assessment in this challenging patient population. In particular, this study seeks to provide a structured longitudinal characterization of diffusion-weighted imaging and ADC evolution and to explore their potential role as imaging biomarkers for monitoring treatment response and infection dynamics in postoperative intracranial infections.

## 2 Material and methods

### 2.1 Study design and objectives

This retrospective study included all consecutive patients treated surgically for postoperative brain abscesses or surgical site infections at the LMU University Hospital between January 2014 and December 2024. The aim was to analyze the clinical course, microbiological spectrum, treatment strategies, and outcomes, with particular emphasis on advanced MRI analyses — including longitudinal imaging with diffusion-weighted imaging (DWI) and quantitative apparent diffusion coefficient (ADC) evaluation — as well as risk factors, antibiotic management, and functional recovery. The primary endpoint of the study was the longitudinal evolution of diffusion-weighted imaging (DWI) and apparent diffusion coefficient (ADC) values as markers of infection activity and treatment response. Secondary endpoints included clinical outcome, laboratory parameters, recurrence requiring repeat surgery, microbiological characteristics, and associations between imaging, laboratory, and clinical parameters.

### 2.2 Patient selection and inclusion criteria

Patients were included if they developed a postoperative brain abscess or surgical site infection (epidural, subdural, or parenchymal) following prior neurosurgical intervention for any indication (e.g., tumor resection, aneurysm clipping, trauma, vascular malformation, or decompressive craniectomy). Inclusion required radiological confirmation (MRI or computed tomography (CT)), microbiological sampling, and available clinical follow-up. Primary (non–surgery-related) infections, incomplete imaging or microbiological data, and missing follow-up led to exclusion. All consecutive eligible patients treated surgically between 2014 and 2024 were included.

### 2.3 Data collection and parameters

**Fig. 1:**
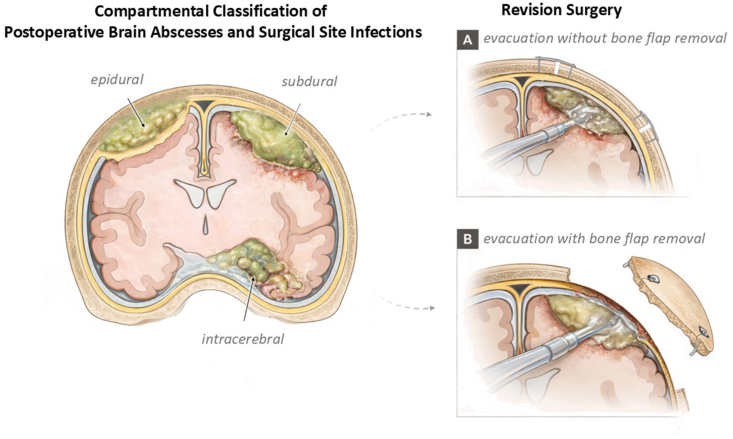
Schematic representation of postoperative surgical site infections, illustrating epidural, subdural, and intracerebral abscesses and empyemas. The figure further depicts the two principal surgical management strategies for infection control: (A) revision surgery without bone flap removal and (B) revision surgery with bone flap explantation.

All data were collected retrospectively from institutional databases and included demographics, surgical history, clinical course, imaging, laboratory, microbiological, and treatment variables. Recorded parameters comprised age and sex, indication, duration of initial surgery, number of index and revision surgeries, and use of foreign materials (PMMA, Lyoplant®, DuraGen®, TachoSil®, CranioFix®, microplates, microscrews). Risk factors for impaired wound healing (clinical wound disorder, purulent discharge, diabetes, smoking, BMI/obesity), prior conservative antibiotic treatment (e.g., a short antibiotic course such as clindamycin), and exposure to radiotherapy, chemotherapy, or immunosuppression were documented. Clinical status and outcomes were assessed using the Glasgow Coma Scale (GCS), Karnofsky Performance Status (KPS), Glasgow Outcome Scale (GOS), and modified Rankin Scale (mRS) pre- and postoperatively. Laboratory markers (CRP, IL-6, leukocytes) were analyzed preoperatively, 3–7 days postoperatively, and at discharge. MRI analyses included T1-weighted sequences before and after application of intravenous contrast agent, T2-weighted imaging, and diffusion-weighted imaging (DWI) including apparent diffusion coefficient (ADC) measurements at clinically defined time points, with anatomical compartmentalization as illustrated in Fig. 1 (epidural vs. subdural/intracerebral involvement). For classification purposes, intracerebral abscesses were assigned to the subdural category. Although intracerebral parenchymal abscesses are located within the brain tissue, they are anatomically situated beneath the dura. This pragmatic decision was made to allow a consistent dichotomous comparison between epidural and subdural infections. The results of the microbiological diagnostics were analyzed with regard to the anatomical location and the number of pathogens detected. Treatment data included antibiotic regimens, total duration of antibiotic therapy, duration of intravenous and oral treatment and antibiotic modifications during the clinical course.

### 2.4 Magnetic resonance imaging

Preoperative and postoperative cranial MRI (cMRI) examinations were available for all patients and were performed either at our institution or at referring hospitals or radiology practices prior to transfer. In cases where imaging was obtained externally, scanner specifications (e.g., magnetic field strength) were not always documented. At our institution, all MRI studies were conducted using 1.5- or 3.0-T scanners (Magnetom Symphony, Siemens, Erlangen, Germany; Signa HDxt, GE Healthcare, Little Chalfont, United Kingdom).

**Fig. 2:**
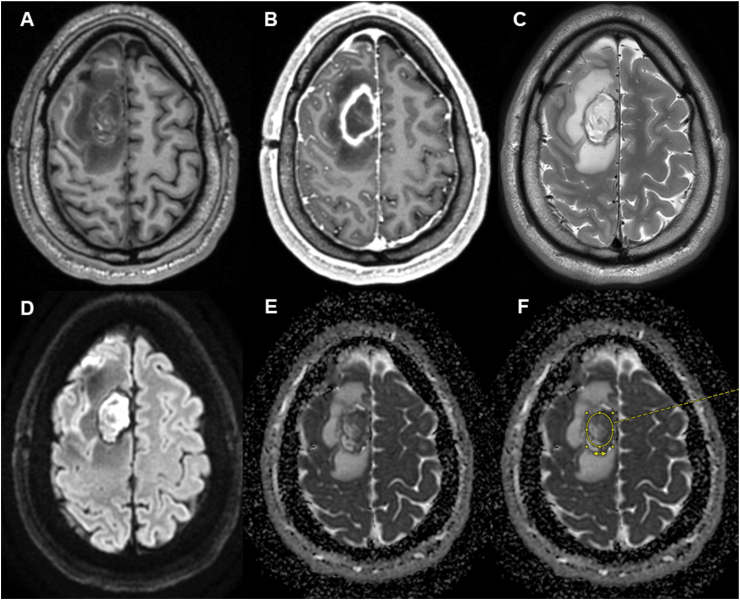
Representative magnetic resonance imaging (MRI) of a postoperative right frontal brain abscess extending into the subdural/intracerebral compartment following glioma resection. (A) Native T1-weighted image demonstrating an iso-/hypointense lesion within the postoperative resection cavity. (B) Contrast-enhanced T1-weighted image showing pronounced ring enhancement consistent with an encapsulated abscess. (C) T2-weighted image depicting a hyperintense cavity with surrounding edema. (D) Diffusion-weighted imaging (DWI) revealing marked diffusion restriction within the abscess cavity. (E) Corresponding apparent diffusion coefficient (ADC) map demonstrating low ADC values in the area of diffusion restriction. (F) ADC map with an exemplary elliptical region of interest (ROI) placed within the abscess cavity for quantitative ADC measurement. The ROI was positioned in regions of maximal signal intensity on the b1000 image and transferred to the co-registered ADC map, with anatomical verification using T2-weighted and DWI sequences, as described in the Methods section.

Standard institutional imaging protocols included axial T2-weighted sequences with a slice thickness of 2 mm and three-dimensional T1-weighted sequences acquired before and after intravenous administration of a macrocyclic gadolinium-based contrast agent (Gadobutrol, Bayer, Leverkusen, Germany. 1.0 mmol/mL; 0.1 mL/kg body weight; 0.1 mmol/kg body weight) (Fig. 2A–C). Each sequence was reconstructed in axial, sagittal, and coronal planes to ensure comprehensive spatial assessment. Particular focus was placed on diffusion-weighted imaging (DWI) including apparent diffusion coefficient (ADC) analysis to characterize diffusion restriction within postoperative collections or abscess cavities (Fig. 2D and E). The DWI protocol applied at our institution consisted of a spin-echo echo-planar imaging (EPI) sequence with the following parameters: repetition time/echo time (TR/TE) = 5700/91 ms, slice thickness = 5 mm with no interslice gap, matrix size = 130 × 130, and field of view (FOV) = 23 cm^2^. ADC maps were automatically generated from b0 and b1000 diffusion data.

For quantitative assessment, a spherical or elliptical region of interest (ROI) was manually placed within the resection cavity, with careful attention to areas exhibiting signal hyperintensity on the b1000 map. The corresponding ROI was then transferred to the co-registered ADC map, ensuring accurate spatial correspondence through linkage by slice position (Fig. 2 F). Correct anatomical placement was verified by cross-referencing T2-weighted and b1000 images. Mean ADC values were extracted from the defined ROIs and used for statistical analyses.

Based on MRI findings, postoperative infections were classified according to their predominant anatomical compartment as either epidural or subdural. In cases where imaging demonstrated involvement of both compartments, infections were categorized as subdural, reflecting the deeper extension of the infectious process and the assumption of progressive contamination from superficial to deeper intracranial compartments in most cases.

**Fig. 3:**
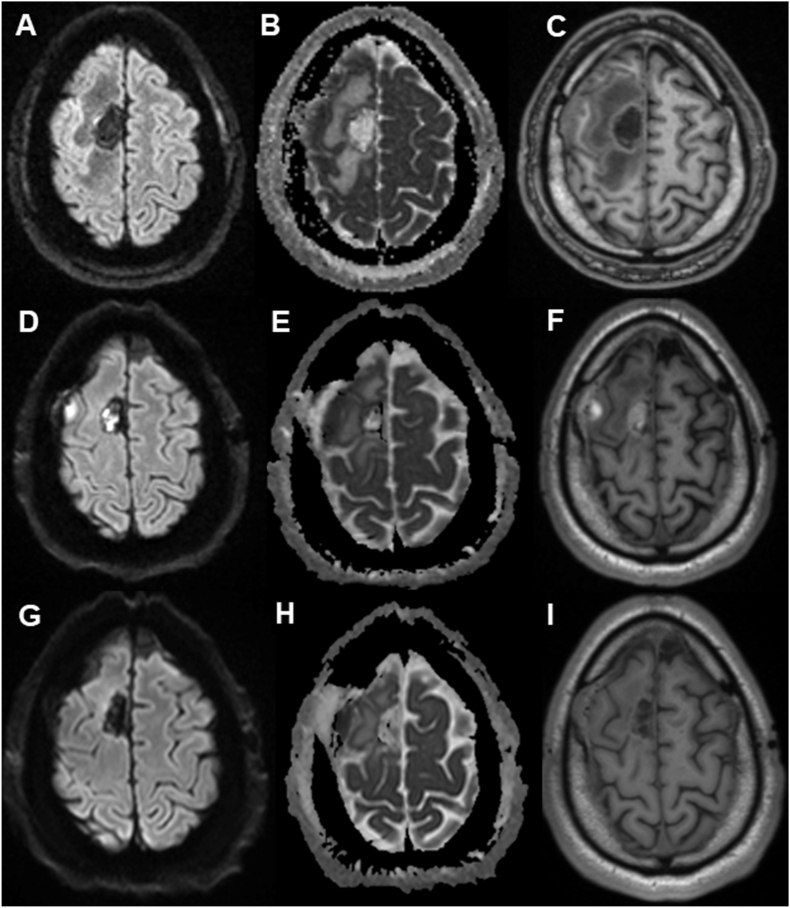
Temporal evolution of a postoperative brain abscess displayed on identical MRI slices using diffusion-weighted imaging (DWI), apparent diffusion coefficient (ADC) maps, and native T1-weighted imaging at different time points. (A–C) Immediate postoperative phase (≤7 days after first infection control surgery): (A) DWI, (B) ADC, (C) native T1-weighted image. (D–F) Early follow-up (4–12 weeks postoperatively): (D) DWI, (E) ADC, (F) native T1-weighted image. (G–I) Final follow-up examination: (G) DWI, (H) ADC, (I) native T1-weighted image.

MRI studies were analyzed at clinically defined time points, including preoperative imaging, the first postoperative MRI obtained within the first 7 days after the initial surgical revision, an early follow-up MRI performed 4–12 weeks postoperatively, and the last available follow-up MRI (Fig. 3).

### 2.5 Surgical interventions

Surgical management was individualized based on clinical and radiological findings and the extent of infection or foreign material involvement, and included revision surgery with or without bone flap explantation (Fig. 1), stereotactic procedures, and in one case intracranial electrode explantation. Stereotactic interventions were planned using iPlan Stereotaxy software (Brainlab, Munich, Germany) based on contrast-enhanced CT scans (0.6-mm slice thickness) co-registered with preoperative MRI (contrast-enhanced T1-weighted, T2-weighted, and contrast-enhanced MR angiography) (Thorsteinsdottir et al., 2025). Stereotactic navigation was performed using the Leksell® Coordinate Frame G (Elekta GmbH, Hamburg, Germany); a 1.0-mm biopsy/aspiration guide tube (inomed Medizintechnik GmbH, Emmendingen, Germany) was inserted through a 2-mm burr hole for targeted abscess access, followed by repeated irrigation with sterile sodium chloride solution for lavage (Schmutzer-Sondergeld et al., 2024a, 2024b).

Bone flaps were explanted whenever infection involved or was in close proximity to the bone flap, including subgaleal infections and epidural or subdural empyemas adjacent to the craniotomy site. In contrast, in cases of deep intracerebral abscesses without radiological or intraoperative evidence of contact with the dura or bone flap, retention of the bone flap was considered, as direct involvement of the osseous structures was not suspected. All revisions were conducted microsurgically with extensive irrigation of the abscess cavity and/or empyema using sterile isotonic sodium chloride solution; purulent material was collected for microbiological analysis and, when indicated, histopathology. Infected or potentially contaminated foreign materials, including dural substitutes (Lyoplant®, DuraGen®), hemostatic agents (TachoSil®), fixation devices (CranioFix®, microplates, microscrews), and polymethylmethacrylate (PMMA) implants, were removed or replaced as appropriate. Subsequent reconstructive procedures, including delayed PMMA cranioplasty, were documented with respect to timing after infection control. Surgical strategy was determined by interdisciplinary neurosurgical consensus based on infection extent, surgical history, microbiological findings, and patient condition.

### 2.6 Statistical analysis

Statistical evaluation was conducted using GraphPad Prism version 8.0 (GraphPad Software, San Diego, CA, USA). Data distribution was assessed prior to analysis to determine the appropriate statistical approach. Continuous variables were compared using Student's t-test for normally distributed data or the Mann–Whitney *U* test for non-normally distributed data. In settings involving more than two groups, particularly in the presence of small or unequal subgroup sizes, continuous variables were analyzed using the non-parametric Kruskal–Wallis test rather than analysis of variance (ANOVA). Categorical variables were examined using the Chi-square test or Fisher's exact test, as appropriate. Time-to-event outcomes, specifically time to recurrence or revision surgery, were evaluated using Kaplan–Meier methodology. Potential predictors of clinical and radiological outcomes were explored through univariate and multivariate regression models. Correlation analyses were performed using Pearson's or Spearman's correlation coefficients depending on variable distribution. All statistical tests were two-sided, and a p-value <0.05 was considered indicative of statistical significance.

### 2.7 Ethical considerations and data protection

The study was approved by the Ethics Committee of LMU Munich (project no. 25-0542; approval 25 July 2025). Data collection complied with data protection regulations, with irreversible anonymization and secure institutional storage. Imaging data were acquired during routine clinical care and analyzed based on finalized neuroradiology reports.

## 3 Results

### 3.1 Patient characteristics

Between 2014 and 2024, 113 patients underwent surgical treatment for postoperative brain abscesses or surgical site infections; 55 were male (48.7%) and 58 female (51.3%), with a mean age of 56.8 ± 15.1 years (males 55.4 ± 16.8 vs. females 58.2 ± 13.3 years; p = 0.3). According to anatomical localization, 56 infections (49.5%) were classified as epidural and 57 (50.5%) as subdural. Infections most commonly followed glioma surgery (n = 42; 37.2%), meningioma resection (n = 25; 22.1%), subdural hematoma evacuation (n = 19; 16.8%), aneurysm clipping (n = 9; 8.0%), and metastasis surgery (n = 7; 6.2%), while other indications accounted for 11 cases (9.7%). Mean body mass index was 25.5 ± 5.3 kg/m^2^, and 18 patients (15.9%) were obese (BMI ≥30 kg/m^2^) (Table 1).

**Table 1:** Baseline demographics, clinical characteristics, imaging findings, and treatment-related parameters of patients with postoperative surgical site infections after neurosurgery, stratified by epidural versus subdural involvement.

*Parameters*	Epidural infection	Subdural infection	*p-value*
**Patient characteristics**			
**Total, n (%)**	56 (49.5)	57 (50.5)	
**Sex, n (%)**			
Male	20 (35.7)	35 (61.4)	**0.008**
Female	36 (64.3)	22 (38.6)	
**Age (years), mean ± SD**	58.8 ± 15.9	55.0 ± 14.2	0.2
**GOS, mean ± SD**			
Preoperative	4.5 ± 0.8	4.2 ± 1.0	**0.04**
Postoperative	4.6 ± 0.8	4.3 ± 1.0	0.07
**mRS, mean ± SD**			
Preoperative	1.6 ± 1.1	1.8 ± 1.0	0.3
Postoperative	1.4 ± 1.3	1.8 ± 1.4	0.2
**KPS, mean ± SD**			
Preoperative	82.0 ± 20.0	77.2 ± 19.1	0.2
Postoperative	83.6 ± 21.4	77.4 ± 23.3	0.1
**Initial surgery, n (%)**			
Subdural hematoma	14 (25.0)	5 (8.8)	**0.02**
Meningioma	7 (12.5)	18 (31.6)	**0.02**
Glioma	20 (35.7)	22 (38.6)	0.8
Aneurysm	6 (10.7)	3 (5.3)	0.3
Metastasis	2 (3.6)	5 (8.8)	0.4
Others	7 (12.5)	4 (7.0)	0.4
**Initial surgical approach, n (%)**			
Craniotomy	52 (92.9)	57 (100.0)	0.06
Burr-hole trepanation	3 (5.3)	0	0.1
Stereotaxy	1 (1.8)	0	0.5
**Foreign materials in initial surgery, n (%)**			
PMMA	6 (10.7)	8 (14.0)	0.8
Lyoplant®	38 (67.9)	42 (73.7)	0.5
DuraGen®	9 (16.1)	11 (19.3)	0.8
TachoSil®	38 (67.9)	46 (80.7)	0.1
CranioFix®	36 (64.3)	43 (75.4)	0.2
Microplates/microscrews	13 (23.2)	11 (19.3)	0.6
**Risk factors for wound healing disorder, n (%)**			
None	43 (76.8)	44 (77.2)	0.99
Diabetes mellitus	4 (7.1)	9 (15.8)	0.2
Nicotine abuse	6 (10.7)	4 (7.0)	0.5
Alcohol abuse	3 (5.4)	0	0.1
Radiotherapy	9 (16.1)	24 (42.1)	**0.004**
Chemotherapy	5 (8.9)	17 (29.8)	**0.008**
Immunosuppressive medication	3 (5.4)	6 (10.5)	0.5
**Wound condition, n (%)**			
Clinical wound healing disorder	40 (71.4)	31 (54.4)	0.08
Discharge of pus	20 (35.7)	22 (38.6)	0.8
**ADC value (x10^−3^mm^2^/s), mean ± SD**			
Preoperative	0.77 ± 0.22	0.77 ± 0.15	0.99
Postoperative (t1)	1.9 ± 0.31	2.0 ± 0.33	0.5
Postoperative (t2)	2.1 ± 0.35	2.2 ± 0.32	0.5
Postoperative (t3)	2.1 ± 0.35	2.5 ± 0.32	**0.004**
**BMI (kg/m^2^), mean ± SD**	25.0 ± 5.3	26.1 ± 5.2	0.3
**CRP (mg/dl), mean ± SD**			
Preoperative	2.4 ± 4.7	5.4 ± 8.2	**0.02**
Postoperative (t1)	3.7 ± 3.7	5.2 ± 6.4	0.1
Postoperative (t2)	1.2 ± 2.8	0.9 ± 1.8	0.5
**IL-6 (pg/ml), mean ± SD**			
Preoperative	24.5 ± 41.1	31.9 ± 90.2	0.6
Postoperative (t1)	26.6 ± 32.5	28.2 ± 28.3	0.8
Postoperative (t2)	5.9 ± 7.1	9.6 ± 23.4	0.3
**Leucocytes (G/l), mean ± SD**			
Preoperative	8.1 ± 3.1	9.3 ± 10.4	0.4
Postoperative (t1)	8.8 ± 3.0	9.5 ± 13.9	0.4
Postoperative (t2)	6.3 ± 2.0	7.4 ± 11.3	0.5
**Duration of antibiotics (days), mean ± SD**			
Total	48.0 ± 25.9	44.1 ± 19.3	0.4
i.v. (intravenous)	28.9 ± 16.8	29.4 ± 18.0	0.9
p.o. (per oral)	24.4 ± 31.7	15.2 ± 15.0	0.05
**Number of changes in antibiotics, mean ± SD**	1.6 ± 0.9	1.6 ± 0.9	0.99
**Follow-Up (months), mean ± SD**	42.3 ± 42.7	30.8 ± 30.5	0.1
**Time to second surgery (days), mean ± SD**	269.0 ± 640.4	91.4 ± 139.1	**0.04**
**Total number of surgeries, mean ± SD**	3.2 ± 0.9	3.2 ± 0.9	0.99
**Duration of initial surgery (min), mean ± SD**	221.8 ± 94.0	243.3 ± 104.9	0.3
***Symptoms***			
**Preoperative symptoms, n (%)**			
None	1 (1.8)	1 (1.8)	0.99
Epileptic seizures	7 (12.5)	8 (14.0)	0.99
Paresis	3 (5.4)	12 (21.1)	**0.02**
Sensory disturbance	2 (3.6)	2 (3.5)	0.99
Infection/sepsis	5 (8.9)	9 (15.8)	0.4
Vertigo	1 (1.8)	2 (3.5)	0.99
Gait disturbance	1 (1.8)	4 (7.0)	0.4
Reduced vigilance	4 (7.1)	10 (17.5)	0.2
Nausea	0	2 (3.5)	0.5
Headache	4 (7.1)	10 (17.5)	0.2
Aphasia	5 (8.9)	3 (5.3)	0.5

ADC = apparent diffusion coefficient; BMI = body mass index; CRP = C-reactive protein; GCS = Glasgow Coma Scale; GOS = Glasgow Outcome Scale; IL-6 = interleukin-6; KPS = Karnofsky Performance Status Scale; mRS = modified Rankin Scale; PMMA = polymethylmethacrylate; SD = standard deviation.

### 3.2 Surgical interventions

Across the cohort, 361 surgical procedures were performed (mean 3.2 per patient). The initial index operation (OP1) in all 113 patients consisted predominantly of craniotomy (n = 109; 96.5%), with burr-hole trepanation (n = 3; 2.6%) and stereotactic surgery (n = 1; 0.9%) being rare. For infection control (OP2), revision with bone flap explantation was required in 83 patients (73.5%), revision without bone flap removal in 29 (25.7%), and electrode explantation in 1 (0.9%). A third surgery (OP3) was performed in 89 patients, most commonly PMMA cranioplasty (n = 52; 58.4%), which was undertaken after confirmed resolution of the infection, followed by revision without (n = 22; 24.7%) or with bone flap removal (n = 13; 14.6%). Fourth surgeries occurred in 33 patients, mainly PMMA plasty (n = 14; 42.4%), revision with bone flap removal (n = 10; 30.3%), or without removal (n = 4; 12.1%). Fifth procedures were required in 10 patients, consisting of revision without bone flap removal (n = 5; 50.0%), PMMA plasty (n = 3; 30.0%), revision with bone flap removal (n = 1; 10.0%), and one stereotactical procedure (n = 1; 10.0%), while sixth surgeries were rare (n = 3), comprising PMMA reconstruction in 2 cases (66.7%) and revision without bone flap removal in 1 case (33.3%).

### 3.3 Diffusion-weighted imaging (DWI) analysis

**Fig. 4:**
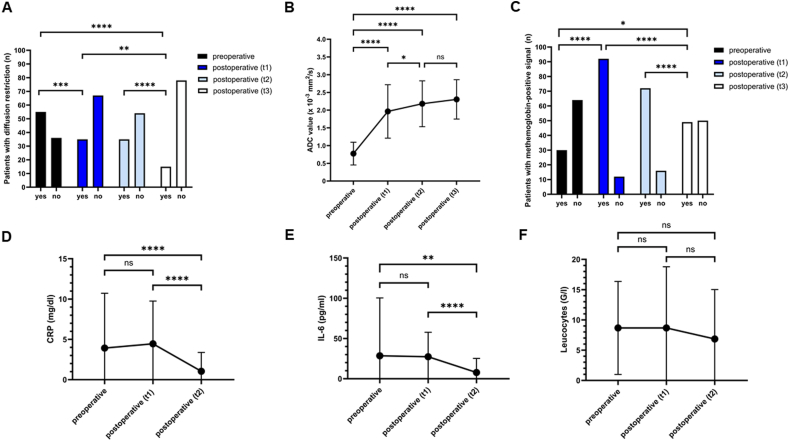
Temporal evolution of imaging and laboratory parameters during the course of postoperative brain abscess. (A) Number of patients with diffusion restriction over time. (B) Longitudinal changes in mean ADC values. (C) Number of patients with methemoglobin-positive signal on native T1-weighted imaging. (D) Temporal course of serum C-reactive protein (CRP) levels. (E) Temporal course of serum interleukin-6 (IL-6) levels. (F) Temporal course of peripheral blood leukocyte counts.

Preoperative MRI was available in 91 patients, with diffusion restriction present in 55 (60.4% of the evaluable cohort). In the early postoperative MRI obtained within the first 7 days postoperatively, diffusion restriction was observed in 35 of 102 patients (34.3%), persisting in 35 of 89 patients (39.3%) at 4–12 weeks and in 15 of 93 patients (16.1%) at last follow-up (Fig. 4A). The proportion of patients demonstrating diffusion restriction significantly decreased from preoperative imaging to early postoperative MRI (p = 0.0003), to the 4–12-week follow-up (p = 0.007), and to the last follow-up (p < 0.0001), with further significant declines from early postoperative to last follow-up (p = 0.005) and from 4 to 12 weeks to last follow-up (p = 0.0005). Stratified by compartment, diffusion restriction was consistently more frequent in subdural than epidural infections but without statistical significance (preoperative: 68.0% vs. 51.2%, p = 0.1; early postoperative: 37.7% vs. 30.6%, p = 0.5; 4–12 weeks: 46.5% vs. 32.6%, p = 0.2; last follow-up: 18.4% vs. 13.6%, p = 0.6).

### 3.4 Apparent diffusion coefficient (ADC) analysis

Mean ADC values increased significantly over time, rising from 0.77 ± 0.32 × 10^−3^ mm^2^/s preoperatively to 1.96 ± 0.75 mm^2^/s in the early postoperative phase (≤7 days), 2.18 ± 0.65 mm^2^/s at 4–12 weeks, and 2.30 ± 0.55 mm^2^/s at last follow-up (Fig. 4B). Significant increases were observed relative to baseline at all postoperative time points (all p < 0.0001). A further significant rise was observed between early postoperative imaging and the 4–12-week follow-up (p = 0.04), whereas the increase between 4 and 12 weeks and last follow-up did not reach statistical significance (p = 0.2). When stratified by infection compartment, ADC values were comparable between epidural and subdural infections at most time points. Preoperatively, no difference was observed (0.77 ± 0.22 vs. 0.77 ± 0.15 × 10^−3^ mm^2^/s; p = 0.99). Similarly, early postoperative ADC values (≤7 days) did not differ significantly (1.9 ± 0.31 vs. 2.0 ± 0.33 mm^2^/s; p = 0.5), nor did intermediate follow-up values at 4–12 weeks (2.1 ± 0.35 vs. 2.2 ± 0.32 mm^2^/s; p = 0.5). At last follow-up, however, subdural infections demonstrated significantly higher ADC values than epidural infections (2.5 ± 0.32 vs. 2.1 ± 0.35 mm^2^/s; p = 0.004).

### 3.5 Methemoglobin (T1 hyperintensity) analysis

T1-weighted MRI demonstrated dynamic methemoglobin-related hyperintensity over time. Preoperatively, 30/94 patients (31.9%) were T1-positive (27 mild, 3 strong), while 64/94 (68.1%) showed no signal. Within the first 7 postoperative days, the frequency of T1 hyperintensity increased to 92/104 patients (88.5%; 64 mild, 28 strong), with 12/104 (11.5%) remaining negative. At 4–12 weeks, positivity persisted in 72/88 patients (81.8%; 70 mild, 2 strong), declining to 49/99 patients (49.5%; 45 mild, 4 strong) at last follow-up (Fig. 4C). Differences between preoperative and all postoperative time points were significant (all p ≤ 0.02), as were declines from early postoperative and 4–12 weeks to last follow-up (both p < 0.0001). Methemoglobin positivity did not differ between epidural and subdural infections at any time point (preoperative: 30.2% vs. 30.3%, p = 0.99; early postoperative: 30.0% vs. 38.6%, p = 0.3; 4–12 weeks: 31.9% vs. 46.8%, p = 0.4; last follow-up: 13.3% vs. 19.6%, p = 0.7).

### 3.6 Correlation of ADC values with methemoglobin, infection compartment, and microbiological and laboratory findings

Associations between ADC and methemoglobin-related T1 hyperintensity were absent at most time points, except for a moderate inverse preoperative correlation (r = −0.36, p = 0.0008) and a weak positive correlation at last follow-up (r = 0.25, p = 0.02). ADC values showed no significant relationship with infection compartment (epidural vs. subdural) preoperatively (r = −0.0007, p = 0.9), at early postoperative imaging (r = 0.07, p = 0.5), or at 4–12 weeks (r = 0.08, p = 0.5). A significant association emerged only at final follow-up (r = 0.3, p = 0.004). Regarding microbiology, early postoperative ADC values correlated inversely with the presence of pathogens in superficial wound swabs (r = −0.46, p = 0.0004), and ADC values at last follow-up correlated inversely with pathogen detection in subdural samples (r = −0.3, p = 0.03).

### 3.7 C-reactive protein (CRP) analysis

Mean CRP was 3.9 ± 6.8 mg/dl preoperatively, 4.5 ± 5.3 mg/dl at 3–7 days postoperatively (p = 0.5), and declined significantly to 1.0 ± 2.3 mg/dl at discharge (vs. preoperative and early postoperative both p < 0.0001), reflecting resolution of the inflammatory reaction over time (Fig. 4D).

### 3.8 Interleukin-6 (IL-6) analysis

Mean IL-6 levels were 28.5 ± 71.9 pg/ml preoperatively and 27.4 ± 30.3 pg/ml at 3–7 days (p = 0.7), decreasing significantly to 7.8 ± 17.5 pg/ml at discharge (vs. preoperative p = 0.004; vs. early postoperative p < 0.0001), reflecting a pronounced attenuation of systemic inflammation by the end of hospitalization (Fig. 4E).

### 3.9 Leukocyte count analysis

Leukocyte counts remained stable (8.7 ± 7.7 G/l preoperative, 8.7 ± 10.1 G/l at 3–7 days, 6.9 ± 8.2 G/l at discharge), with no significant differences between time points (preoperative vs. early postoperative p = 0.8; preoperative vs. discharge p = 0.07; early postoperative vs. discharge p = 0.09). This lack of significant variation may be attributable to the nonspecific nature of leukocytes, which can be influenced by a wide range of physiological and inflammatory conditions (Fig. 4F).

### 3.10 Surgical materials

During the initial neurosurgical procedure prior to infection onset, various surgical adjuncts and materials were used according to intraoperative findings and reconstruction requirements. Polymethylmethacrylate (PMMA) was applied in 14 cases (12.4%), Lyoplant® in 80 cases (70.8%), DuraGen® in 20 cases (17.7%), TachoSil® in 84 cases (74.3%), CranioFix® in 79 cases (69.9%), and microplates or microscrews in 24 cases (21.2%).

### 3.11 Risk factors for subdural extension of postoperative infection

In this study, we further distinguished postoperative infections according to their anatomical localization, differentiating between epidural and subdural compartments (Fig. 1). Intracerebral abscesses were included within the subdural category, as they are anatomically located beneath the dura, and were therefore considered part of the subdural compartment for analytical consistency. Within our cohort, 49.5% of infections were classified as epidural and 50.5% as subdural, allowing for a more nuanced analysis of radiological and clinical characteristics across these two entities.

Univariate analyses identified elevated preoperative CRP levels, the presence of multiple pathogens, prior radiotherapy, and prior chemotherapy as factors associated with radiological extension of infection into the subdural compartment. In contrast, preoperative IL-6 levels, leukocyte counts, diffusion restriction, methemoglobin signal characteristics, and ADC values were not significantly associated with compartmental extension.

After adjustment in multivariate analysis, only preoperative CRP levels (p = 0.009) and the presence of multiple pathogens (p = 0.02) remained independently associated with subdural involvement, whereas the effects of prior radiotherapy and chemotherapy were attenuated and did not retain statistical significance. These findings suggest that systemic inflammatory burden and microbiological complexity represent the primary independent factors associated with subdural extension, while treatment-related variables may act as contributing but not independent predictors.

### 3.12 Antibiotic treatment characteristics

Antibiotic treatment was comparable between epidural and subdural infections, with similar total duration (48.0 ± 25.9 vs. 44.1 ± 19.3 days, p = 0.4), intravenous therapy (28.9 ± 16.8 vs. 29.4 ± 18.0 days, p = 0.9), and number of regimen changes (both 1.6 ± 0.9, p = 0.99), while oral therapy tended to be longer in epidural infections (24.4 ± 31.7 vs. 15.2 ± 15.0 days, p = 0.05). Follow-up duration was longer in epidural cases (42.3 ± 42.7 vs. 30.8 ± 30.5 months, p = 0.1). Time to second surgery was significantly prolonged in epidural compared with subdural infections (269.0 ± 640.4 vs. 91.4 ± 139.1 days, p = 0.04), suggesting differences in disease progression or clinical urgency, whereas total surgery number and index procedure duration did not differ (p = 0.99 and p = 0.3).

### 3.13 Risk factors for wound healing disorders

Diabetes mellitus (4/56, 7.1% vs. 9/57, 15.8%; p = 0.2), nicotine abuse (6/56, 10.7% vs. 4/57, 7.0%; p = 0.5), alcohol abuse (3/56, 5.4% vs. 0; p = 0.1), and immunosuppressive medication (3/56, 5.4% vs. 6/57, 10.5%; p = 0.5) did not differ significantly between groups (epidural infection vs subdural infection). In contrast, radiotherapy (9/56, 16.1% vs. 24/57, 42.1%; p = 0.004) and chemotherapy (5/56, 8.9% vs. 17/57, 29.8%; p = 0.008) were more frequent in subdural infections.

### 3.14 Wound condition and purulent discharge

Clinically evident wound healing disorders were common and tended to be more frequent in epidural infections than in subdural infections (40/56, 71.4% vs. 31/57, 54.4%; p = 0.08), while purulent discharge occurred at similar rates (20/56, 35.7% vs. 22/57, 38.6%; p = 0.8), indicating that overt pus formation was not specific to infection localization.

### 3.15 Comparison of preoperative and postoperative symptoms

Clinical symptoms improved significantly after treatment: asymptomatic patients increased from 2/113 (1.8%) preoperatively to 83/113 (73.5%) postoperatively (p < 0.0001). Wound healing disorders resolved completely (71/113, 62.8% vs. 0%, p < 0.0001), infection/sepsis decreased from 14/113 (12.4%) to 1/113 (0.9%, p = 0.0007), and headache from 14/113 (12.4%) to 1/113 (0.9%, p = 0.0007). Other neurological symptoms (epileptic seizures, paresis, reduced vigilance, aphasia) improved numerically but not significantly (all p > 0.05) (Table 2).

**Table 2:** Preoperative and postoperative neurological and systemic symptoms in patients with postoperative surgical site infections and abscesses.

*Symptoms*	Preoperative, n (%)	Postoperative, n (%)	*p-value*
None	2 (1.8)	83 (73.5)	**<0.0001**
Epileptic seizures	15 (13.3)	9 (8.0)	0.3
Paresis	15 (13.3)	10 (8.8)	0.4
Sensory disturbance	4 (3.5)	0	0.1
Infection/sepsis	14 (12.4)	1 (0.9)	**0.0007**
Vertigo	3 (2.7)	0	0.2
Gait disturbance	5 (4.4)	2 (1.8)	0.4
Reduced vigilance	14 (12.4)	8 (7.1)	0.3
Nausea	2 (1.8)	1 (0.9)	0.99
Headache	14 (12.4)	1 (0.9)	**0.0007**
Aphasia	8 (7.1)	6 (5.3)	0.8
Wound healing disorder	71 (62.8)	0	**<0.0001**

### 3.16 Pathogen spectrum across anatomical compartments

Microbiological analysis showed high pathogen detection across compartments, with monomicrobial infections predominating, especially in subdural infections (40/57, 70.2% vs. 24/56, 42.9% in epidural; p = 0.004), while polymicrobial infections (≥2 pathogens) were more common in epidural cases without significance (Table 3). Overall, 160 isolates representing 21 pathogens were identified, and no pathogen was detected in 7/113 cases (Table 4).

**Table 3:** Microbiological swab results across different anatomical compartments stratified by epidural and subdural infections.

	Epidural infection	Subdural infection	*p-value*
**Number of pathogens, n (%)**			
0	5 (8.9)	2 (3.5)	0.3
1	24 (42.9)	40 (70.2)	**0.004**
2	19 (33.9)	11 (19.3)	0.09
3	8 (14.3)	4 (7.0)	0.2
***Superficial wound***			
**Detection of pathogens**			
Not performed	22 (39.3)	23 (40.4)	0.99
Yes	29 (51.8)	31 (54.4)	0.9
No	5 (8.9)	3 (5.3)	0.5
***Subcutaneous/subgaleal***			
**Detection of pathogens**			
Not performed	11 (19.6)	10 (17.5)	0.8
Yes	42 (75.0)	42 (73.7)	0.99
No	3 (5.4)	5 (8.8)	0.7
***Epidural***			
**Detection of pathogens**			
Not performed	12 (21.4)	3 (5.3)	**0.01**
Yes	40 (71.4)	49 (86.0)	0.07
No	4 (7.1)	5 (8.8)	0.99
***Subdural***			
**Detection of pathogens**			
Not performed	24 (42.9)	23 (40.4)	0.8
Yes	25 (44.6)	29 (50.9)	0.6
No	7 (12.5)	5 (8.8)	0.6
***Subarachnoidal***			
**Detection of pathogens**			
Not performed	50 (89.3)	54 (94.7)	0.3
Yes	6 (10.7)	3 (5.3)	0.3
No	0	0	0.99

**Table 4:** Microbiological spectrum of postoperative surgical site infections and abscesses. A total of 160 microbial isolates representing 21 distinct pathogens were identified across the cohort; no pathogen was detected in 7 cases. The number of detections (n) and corresponding percentages (%) are reported for each pathogen.

Pathogen	n	%
Cutibacterium acnes	53	33.1%
coagulase-negative Staphylococci	43	26.9%
*Staphylococcus aureus*	30	18.8%
*E. coli*	5	3.1%
*Pseudomonas aeruginosa*	4	2.5%
Enterobacter spp.	3	1.9%
Klebsiella spp.	3	1.9%
Enterococcus spp.	2	1.3%
*Citrobacter koseri*	2	1.3%
*Peptoniphilus indolicus*	2	1.3%
*Finegoldia magna*	2	1.3%
Candida spp.	2	1.3%
Streptococcus spp.	1	0.6%
*Corynebacterium afermentans*	1	0.6%
*Actinomyces turicensis*	1	0.6%
Anaerococcus spp.	1	0.6%
*Veillonella parvula*	1	0.6%
Salmonella typhimurium	1	0.6%
*Serratia liquefaciens*	1	0.6%
*Stenotrophomonas maltophilia*	1	0.6%
Aspergillus spp.	1	0.6%

Superficial wound and subcutaneous/subgaleal cultures were positive in approximately ∼50–75% of both groups; epidural compartment cultures were more often positive in subdural infections (49/57, 86.0% vs. 40/56, 71.4%), whereas subdural compartment cultures were positive in ∼45–51% without group differences.

### 3.17 Time to recurrence, recurrence-free survival and risk factors for recurrence

**Fig. 5:**
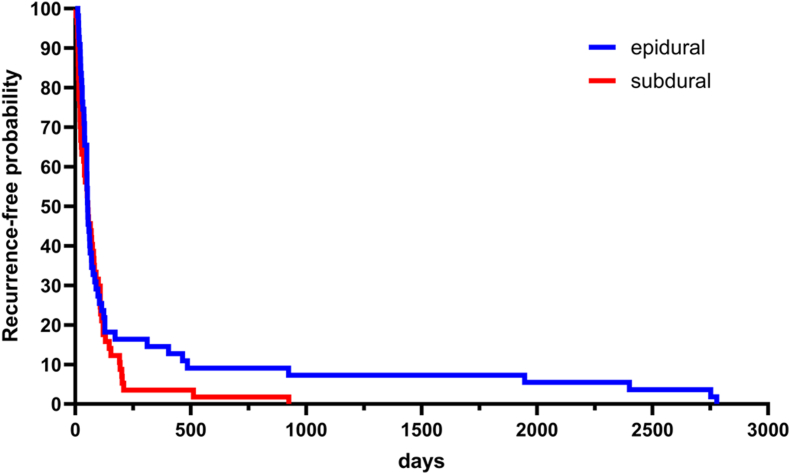
Kaplan–Meier analysis of recurrence-free probability showing the time to first abscess/empyema surgery after the initial neurosurgical procedure. Curves are stratified by epidural and subdural infection. No significant difference in recurrence-free survival was observed between groups (log-rank test, p = 0.2).

Time to recurrence requiring repeat surgery was significantly longer in epidural infections (269.0 ± 640.4 days) than in subdural infections (91.4 ± 139.1 days; p = 0.04), as illustrated by Kaplan–Meier analysis of recurrence-free probability stratified by infection compartment (Fig. 5). Notably, 50.9% of all recurrences occurred within 53 days after the initial neurosurgical procedure in both epidural and subdural infection compartments. Interestingly, comparison of early recurrences (≤53 days) and late recurrences (>53 days) revealed no significant differences in the underlying pathogen spectrum. In univariate analysis, the use of fixation materials (CranioFix® and microplates/microscrews, both p = 0.02) as well as lower preoperative ADC values (p = 0.03) were associated with recurrence. However, in multivariate analysis, none of these factors remained statistically significant, indicating that recurrence is likely influenced by multiple interacting factors rather than single independent predictors.

## 4 Discussion

In this retrospective cohort study of 113 patients treated surgically for postoperative brain abscesses and surgical site infections, we combined detailed clinical, microbiological, and laboratory data with longitudinal MRI analyses, with a particular focus on diffusion-weighted imaging (DWI), apparent diffusion coefficient (ADC) dynamics, and temporal signal evolution across follow-up. This approach builds on our previous work in primary brain abscesses, where we demonstrated – using serial MRI volumetrics – that structured longitudinal imaging provides valuable insight into abscess evolution and treatment response across different surgical strategies (Harapan et al., 2026). Although the present study does not directly alter established surgical or antibiotic management strategies, it provides clinically relevant insights into the interpretation of postoperative MRI. The identification of reproducible DWI/ADC trajectories may support differentiation between persistent infection and resolving postoperative changes, thereby contributing to more informed clinical decision-making during follow-up.

The primary findings can be summarized as follows: (1) surgical revision combined with prolonged antibiotic therapy resulted in marked clinical recovery, with significant resolution of presenting symptoms and normalization of systemic inflammatory markers; (2) recurrence patterns demonstrated a clinically relevant temporal clustering, with approximately half of all repeat surgeries occurring within the early postoperative period (≤53 days), while time to recurrence differed between infection compartments (recurrence was shorter in subdural infections compared with epidural infections); (3) microbiological analyses revealed a heterogeneous pathogen spectrum with high detection rates across anatomical compartments, and microbiological complexity (presence of multiple pathogens) emerged as an independent factor associated with subdural involvement; (4) longitudinal MRI demonstrated a progressive attenuation of diffusion restriction accompanied by a robust and sustained increase in ADC values across follow-up, consistent with resolving diffusion restriction; (5) epidural and subdural infections exhibited broadly comparable diffusion characteristics at most time points, with compartment-specific differences becoming evident primarily at later stages; and (6) diffusion-related MRI parameters predominantly reflected inflammatory activity rather than hemorrhagic confounders, as supported by the absence of stable associations with methemoglobin signal alterations.

Overall clinical outcomes in this cohort were favorable, with a marked reduction in infection-related symptoms and a substantial increase in asymptomatic patients at follow-up. The resolution of wound healing disorders, infection or sepsis, and headache after treatment underscores the effectiveness of combined surgical revision and prolonged antimicrobial therapy, which remains the standard of care in postoperative intracranial infections (Hakan, 2008). These findings are consistent with prior reports describing satisfactory outcomes when prompt surgical source control is combined with targeted antibiotics, even in complex postoperative settings (Lange et al., 2018; Cavusoglu et al., 2008; Eibl et al., 2025).

A central aspect of this study is the systematic longitudinal analysis of MRI findings across multiple time points. Diffusion restriction on DWI was common preoperatively and declined in the postoperative course, with a continuous decrease observed through early postoperative imaging, intermediate follow-up (4–12 weeks), and final follow-up. This temporal pattern aligns with established concepts of restricted diffusion as a marker of purulent material, high cellularity, and viscous abscess content, which gradually resolve with effective drainage and antimicrobial therapy (Cartes-Zumelzu et al., 2004).

Quantitative ADC analysis complemented these qualitative observations. Mean ADC values increased significantly from preoperative to postoperative time points and continued to rise at later follow-up. This pattern is physiologically plausible, as abscess resolution is typically associated with reduced cellularity, liquefaction of purulent material, and restoration of extracellular water mobility, resulting in higher diffusivity. In the postoperative setting, this increase likely reflects progressive clearance of infection and decreasing inflammatory viscosity rather than confounding postoperative tissue effects. Previous studies on non-postoperative brain abscesses have shown that these lesions are typically characterized by high signal on DWI and low ADC values, reflecting marked diffusion restriction caused by viscous purulent content, high cellularity, and inflammatory debris. This supports the role of ADC as a biomarker of abscess biology when interpreted in the appropriate longitudinal clinical context (Chang et al., 2002; Moradi et al., 2025).

Our diffusion findings are consistent with previous work demonstrating the robustness of DWI/ADC for detecting postoperative intracranial abscesses and proposing an ADC cut-off of 1.87 × 10^−3^ mm^2^/s to distinguish abscess from normal postoperative changes (Schwartz et al., 2019). In line with this, preoperative ADC values in our cohort were clearly below this threshold (epidural 0.77 ± 0.22; subdural 0.77 ± 0.15 × 10^−3^ mm^2^/s), supporting the presence of true abscess activity and/or empyema. In contrast, postoperative ADC values at all follow-up time points exceeded the 1.87 cut-off in both epidural and subdural infections, paralleling the decline in diffusion restriction and indicating transition away from highly cellular, purulent cavities.

A further relevant finding concerns diffusion differences between compartments. ADC values did not differ between epidural and subdural infections preoperatively or at early/intermediate postoperative imaging (all p ≥ 0.5). Only at final follow-up did subdural infections show significantly higher ADC values than epidural infections (2.5 ± 0.32 vs. 2.1 ± 0.35, p = 0.004). Persistent structural confinement in epidural collections may maintain relatively lower diffusivity, whereas the more expansive subdural space may permit greater fluid redistribution and reduction of cellular debris over time, resulting in higher late ADC values. These interpretations remain speculative but indicate that longitudinal diffusion behavior may differ between compartments mainly in the chronic stage.

Prior radiotherapy was frequent in our cohort and significantly more common in subdural infections, consistent with prior studies identifying radiotherapy as a risk factor for surgical site infections after craniotomy due to impaired tissue healing and local immune response (Lee et al., 2024).

The microbiological findings of this cohort are consistent with existing literature on postoperative intracranial infections, showing high pathogen detection rates and a predominance of monomicrobial infections (Lange et al., 2018; Carone et al., 2025). The higher frequency of polymicrobial infections in epidural cases, although not statistically significant, may reflect greater exposure to skin microbiome and superficial contamination. Importantly, no pathogen was identified in a small subset of patients, highlighting the known limitations of microbiological diagnostics in patients who may have received prior antibiotic therapy. The high prevalence of Cutibacterium acnes requires careful interpretation, as this organism is part of the normal skin flora and may represent either contamination or true infection (Chiang et al., 2011). In the postoperative neurosurgical setting, however, C. acnes is increasingly recognized as a relevant pathogen(Jimenez-Martinez et al., 2019), particularly in implant-associated infections where biofilm formation plays a crucial role (Coenye et al., 2022). Biofilm-associated infections on foreign materials such as bone flaps, fixation devices, or dural substitutes may contribute to persistent or recurrent infection and often necessitate surgical removal of the affected material (Jallo et al., 1999).

Recurrence requiring repeat surgery occurred relatively early in many cases, with approximately half of all recurrences occurring within the first two months. The significantly longer time to recurrence in epidural infections compared with subdural infections may reflect differences in disease progression and thresholds for reoperation. An explanation is that epidural infections may remain clinically silent for longer periods, as they do not directly involve the brain parenchyma and therefore may cause fewer or less severe neurological symptoms. In contrast, subdural infections may be closer to eloquent cortical structures and can lead to earlier and more pronounced clinical deterioration, prompting earlier reoperation. Moreover, deeper subdural involvement may reflect a higher microbial burden or more advanced inward spread of infection, which could necessitate earlier surgical intervention.

While several clinical and microbiological findings confirm known patterns, this study extends existing knowledge by providing a systematic longitudinal analysis of diffusion dynamics across predefined time points. The structured assessment of DWI/ADC evolution in this cohort represents one of the most comprehensive analyses in postoperative intracranial infections to date. Overall, these findings define a consistent temporal pattern of diffusion normalization, characterized by decreasing diffusion restriction and increasing ADC values, which may support interpretation of follow-up MRI and help differentiate resolving postoperative changes from persistent or recurrent infection.

This study has several important limitations that must be acknowledged. First, its retrospective design is subject to selection bias, missing data, and heterogeneity in clinical decision-making. In addition, the single-center nature of the cohort may limit the generalizability of the findings to other institutions with different patient populations and treatment strategies. Second, MRI examinations were performed on different scanners, including external institutions, with variable field strengths and acquisition parameters, which may have influenced quantitative ADC measurements despite standardized ROI placement. However, we believe that these differences are rather negligible, as it has been shown that ADC values are highly reproducible independent of MRI hardware (Ogura et al., 2015; Belli et al., 2016; Grech-Sollars et al., 2015). Third, the classification of intracerebral abscesses within the subdural category represents a pragmatic simplification for analytical purposes and may not fully reflect anatomical and pathophysiological differences between compartments. Fourth, we did not assess the potential impact of extracellular methemoglobin due to the heterogenous dataset. In general, hemorrhagic changes after brain surgery may yield increased T1 and consequently false-positive DWI signal which in turn may be misinterpreted as (residual) abscess. Fifth, follow-up intervals were not uniform, and imaging time points were defined clinically rather than prospectively, introducing temporal variability. Microbiological data may have been affected by prior antibiotic exposure, and laboratory markers were not available at all time points for all patients. Finally, although this represents one of the larger single-center cohorts with longitudinal diffusion imaging in postoperative intracranial infections, the sample size may still be insufficient to detect smaller subgroup effects, particularly in multivariate analyses of recurrence, where the number of events limits the identification of independent predictors.

In summary, the central contribution of this study lies in the integration of longitudinal quantitative diffusion imaging with clinical and microbiological data in a relatively large cohort of patients with postoperative intracranial infections, enabling a structured characterization of postoperative infection dynamics beyond purely descriptive analyses. This study provides a longitudinal characterization of diffusion dynamics in postoperative intracranial infections and identifies ADC evolution as a reproducible imaging biomarker that may support objective monitoring of treatment response beyond conventional clinical and laboratory parameters. While the retrospective design limits causal interpretation, the integrated imaging approach represents a pragmatic contribution to the understanding of postoperative infection dynamics in routine clinical practice.

## 5 Conclusions

This study provides a comprehensive characterization of postoperative brain abscesses and surgical site infections after neurosurgery, integrating clinical characteristics, underlying risk profiles, microbiological findings, and longitudinal DWI/ADC MRI analysis. Postoperative surgical site infections demonstrated distinct etiological patterns, frequent need for repeated surgical intervention, and a microbiological spectrum dominated by staphylococci and Gram-negative pathogens, often in polymicrobial constellations. Systemic inflammatory markers and neurological status improved under combined surgical and prolonged targeted antibiotic therapy. Serial DWI and ADC measurements proved valuable for objectively tracking infection dynamics, with decreasing diffusion restriction and rising ADC values reflecting therapeutic response over time. The structured longitudinal assessment of DWI and ADC may serve as a practical adjunct for treatment monitoring and may improve interpretation of postoperative imaging in complex neurosurgical infection scenarios. Our results highlight the importance of structured clinical assessment, risk-adapted management, microbiology-guided therapy, and longitudinal diffusion imaging in the care of postoperative intracranial infections. Prospective multicenter studies with standardized imaging protocols are warranted to further establish diffusion metrics as biomarkers for treatment monitoring and recurrence risk.

## Declaration of competing interest

The authors declare that they have no known competing financial interests or personal relationships that could have appeared to influence the work reported in this paper.
